# Alleviating sepsis: Revealing the protective role of costunolide in a cecal ligation and puncture rat model 

**DOI:** 10.22038/IJBMS.2024.75372.16335

**Published:** 2024

**Authors:** Mustafa Can Güler, Ayhan Tanyeli̇, Ersen Eraslan, Özgür Çelebi̇, Demet Çelebi̇, Selim Çomakli, Emir Enis Yurdgülü, Yasin Bayir

**Affiliations:** 1 Department of Physiology, Faculty of Medicine, Atatürk University, Erzurum, Turkey; 2 Department of Physiology, Faculty of Medicine, Bandırma OnYedi Eylül University, Balıkesir, Turkey; 3 Department of Medical Microbiology, Faculty of Medicine, Ataturk University, Erzurum, Turkey; 4 Department of Microbiology, Faculty of Veterinary Medicine, Ataturk University, Erzurum, Turkey; 5 Ataturk University Vaccine Application and Development Center, Ataturk University, Erzurum, Turkey; 6 Department of Pathology, Faculty of Veterinary, Atatürk University, Erzurum, Turkey; 7 Department of Biochemistry, Faculty of Pharmacy, Atatürk University, Erzurum, Turkey

**Keywords:** Cecal ligation and puncture, Costunolide, Oxidative stress, Rat, Sepsis

## Abstract

**Objective(s):**

Sepsis poses a significant threat to human life, rendering it a burdensome medical disease. Despite significant advancements, the current state of medical science still lacks a viable and efficacious cure. Costunolide (COST) is a multifaceted sesquiterpene lactone that exhibits a range of actions, including anti-inflammatory and antioxidant properties. We investigated the potential impacts of COST on a rat sepsis model caused by cecal ligation and puncture (CLP).

**Materials and Methods:**

We created an experimental rat model with the following groups: SHAM, CLP, CLP+low dose COST, and CLP+high dose COST. Blood, kidney, and lung samples were collected. Inflammatory mediators such as interleukin-1beta (IL-1β), IL-6, tumor necrosis factor-alpha (TNF- α), and nuclear factor kappa-B (NF-κB) were investigated. In addition, we assessed oxidative stress by measuring 8-Hydroxydeoxyguanosine (8-OHdG) immunopositivity, MDA levels, glutathione (GSH), and superoxide dismutase (SOD) activity. Histopathological and immunohistochemical examinations backed up our findings.

**Results:**

Compared to the CLP group, the COST group showed a reduction in inflammatory and oxidative stress indicators. The expression of inflammatory mediators was suppressed by COST, and histological examinations revealed improvements in kidney and lung tissues in the treatment groups.

**Conclusion:**

Our study highlights the preventive effects of COST against CLP-induced sepsis-related injury. Considering its beneficial effects against many diseases, COST is worthy as to be evaluated against sepsis.

## Introduction

Sepsis is a life-threatening inflammatory reaction caused by infections (1). It is the uncontrolled host response through inflammatory and immune processes against microbial invasions (2). Sepsis is a complicated condition with acute organ malfunction and a high mortality risk (3). It is a significant factor in enhancing mortality among hospitalized patients (4) with critical diagnoses (5). There are approximately 50 million sepsis cases and 11 million sepsis-related deaths in the world annually (6). Furthermore, severe COVID-19 cases have developed septic shock accompanied by inflammatory storms and many deaths due to sepsis (7). In addition, sepsis leads to multiple organ dysfunction worsening the course and contributing to mortality (8). The kidneys and lungs are two of the organs that are commonly damaged by sepsis (9). 

High reactive oxygen species (ROS) levels generally damage the human body (10). ROS act as a second messenger and affect unfavorably signal cascades (11). ROS overproduction results in cytokine release, leukocyte infiltration, and lipid peroxidation which can cause oxidative stress and organ damage (12). Sepsis induces oxidative stress (13). The formation of ROS is enhanced by proinflammatory cytokines, such as interleukin-6 (IL-6) and tumor necrosis factor-alpha (TNF-α), contributing to organ harm associated with sepsis (14). The overabundance of cytokines recruits macrophages and neutrophils to the site of infection, where they subsequently release cytotoxic ROS, intensifying the immunological response (15). Several parameters contribute to evaluating oxidative stress. 8-Hydroxydeoxyguanosine (8-OHdG) is a biomarker that is commonly used to assess oxidative DNA damage (16). Malondialdehyde (MDA) is used for the assessment of lipid peroxidation (17). Glutathione (GSH) inhibits oxidative stress, as a part of the anti-oxidative system (18). In addition, superoxide dismutase (SOD) is a vital antioxidant enzyme against ROS activity (19).

Sepsis is characterized by an inflammatory response triggered by infections, leading to the release of proinflammatory cytokines such as interleukin-1beta (IL-1β), IL-6, and TNF-α (20). TNF-α, IL-6, and IL-1β play a pivotal role in the onset of the systemic inflammatory response (21). Nuclear factor kappa-B (NF-κB) regulates immunological modulation and other inflammatory response-related functions (22). The NF-κB transcription factor orchestrates the synthesis of many molecules, such as IL-1β, IL-6, and TNF-α, playing a significant role in the initiation and progression of the inflammatory response (23). Sepsis triggers multiple organ dysfunction through the inflammatory mechanisms (24). The exacerbation of sepsis, resulting in consequences such as multiple organ failure, is attributed to the detrimental effects of inflammatory cytokines, including IL-6 and TNF-α (25). 

**Figure 1:**
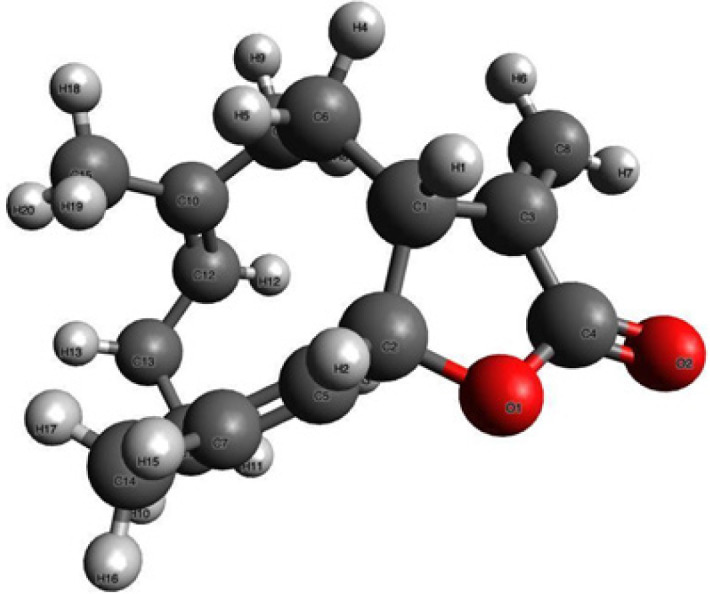
3D chemical structure of COST (Created with Avogadro version 1.2.0., http://avogadro.cc/)

Costunolide (COST, C_15_H_20_O_2_, Figure 1), a sesquiterpene lactone, is widely available in several plant families and is used in traditional East Asian medicine for treating infectious and inflammatory diseases (26). COST demonstrates antioxidant, anti-inflammatory, and antibiotic activities (27). Besides, it has additional effects like anticancer properties (28). As we mentioned, COST has been examined in various conditions, but not in sepsis. 

We hypothesized that the features we mentioned above made COST an appropriate candidate to examine the potential effects on cecal ligation and puncture (CLP)-induced sepsis model in rats. There are several sepsis models in the literature (29). We preferred the CLP-induced sepsis model because it is quite similar to sepsis in humans (30) in terms of the resemblance to perforated appendicitis or perforated diverticulitis (31). 

To support our hypothesis, we assessed vital inflammatory markers, such as TNF-α, IL-6, NF-κB, and IL-1β. Besides we examined 8-OHdG as an oxidative DNA damage marker. We wanted to support the results with SOD, MDA, and GSH parameters. In addition, we investigated tissue samples with histological examination to compare with the other findings. In this way, we hoped to improve the sepsis-induced multiple organ injury treatment in terms of morbidity and mortality. 

## Materials and Methods

***Ethical Approval***

Atatürk University Local Ethics Council of Animal Experiments confirmed the study (Registration Number: 75296309-050.01.04-E.2000143516, Approved Protocol Number: 11/06/2020-95). The experiment was performed at Atatürk University Medical Experimental Application and Research Center (MEARC). We carried out the present study in compliance with the existing protocols of the ethics committee and the Helsinki Declaration of the World Medical Association recommendations on animal studies. 

***Experimental animals***

MEARC provided 40 female Wistar Albino rats (12–16 weeks old, weighing 200–250 g) and a surgical room for the experimental procedure. The animals were housed in MEARC with a 12 hr light/dark cycle, 50–55% humidity at 22–25 °C, and granted food and water access *ad libitum*. They were allowed to get used to the environment for ten days before the study.

***Chemicals***

We procured a 10% povidone-iodine solution (Batticon; Adeka) for disinfection, and xylazine hydrochloride (Rompun®, Bayer, Istanbul) and ketamine (Ketalar®, Pfizer, Istanbul) for anesthesia. We purchased COST (purity ≥95%, CAS: 553-21-9) from TCI America (USA) and stored it (-20 °C, sealed storage, away from moisture and light) until the experimental procedure. COST was dissolved in 5% dimethyl sulfoxide (DMSO) and 10% Tween-20 in phosphate buffer solution (PBS) before administration. The application doses (5 mg/kg and 10 mg/kg) were based on and modified from previous animal studies (32, 33). 

***CLP model***

**Figure 2:**
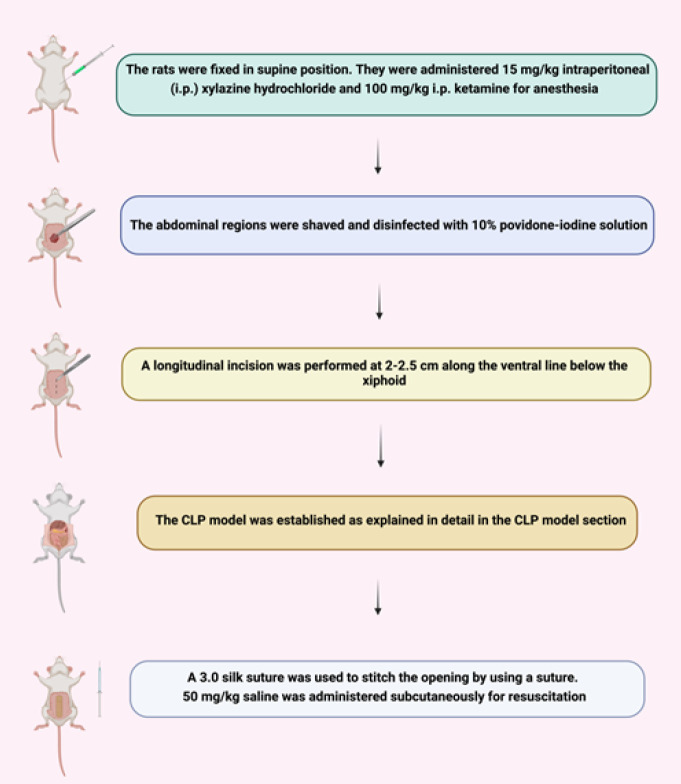
Summary of the CLP model process (created with BioRender.com)

**Figure 3:**
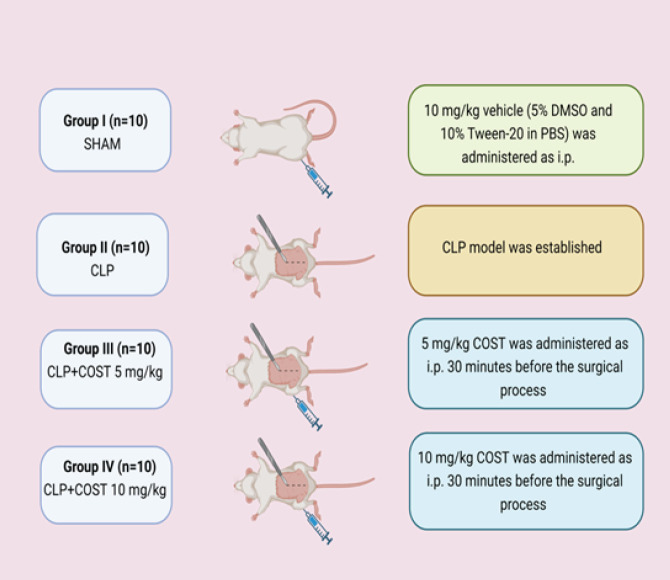
Design of the experimental study groups (created with BioRender.com)

Figure 2 summarizes the preoperative preparation and the CLP model process. First, the rats were immobilized in the supine position. 15 mg/kg intraperitoneal (IP) xylazine hydrochloride and 100 mg/kg IP ketamine were administered for anesthesia (34). The abdominal regions were shaved and disinfected with 10% povidone-iodine solution. Four study groups were designed randomly (Figure 3).

Group I (SHAM, n=10): A longitudinal incision at 2–2.5 cm along the ventral line below the xiphoid was performed. The abdominal cavities of the animals were opened and closed back. The vehicle (5% DMSO and 10% Tween-20 in PBS) was IP administered as 10 mg/kg. 

**Figure 4:**
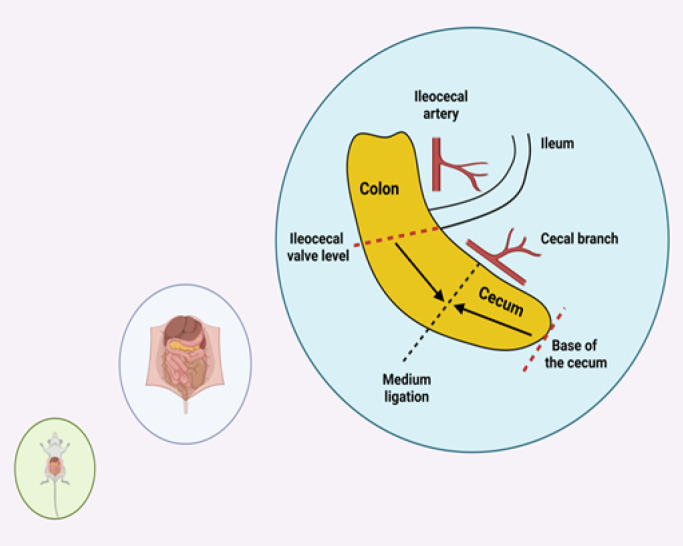
Establishment of the mid-grade sepsis through a medium ligation. Equal ligation distances between the base of the cecum and the ileocecal valve level (created with BioRender.com)

Group II (CLP, n=10): A CLP model was established from previous studies (35, 36). We arrived in the ventral cavity after the abdominal incision. The cecum was located and externalized. We gently dissected the cecum’s mesentery to prevent damaging the ileocecal artery’s cecal branch. The severity of the CLP model is influenced not only by sepsis duration and needle size but also by cecum ligation length (37). To cause mid-grade sepsis, we did a medium ligation (Figure 4). The distance from the ligation to the base of the cecum and the distance between the distal pole and the ligation were essentially the same. We tied the distal cecum below the ileocecal valve level to avoid intestinal obstruction. Then, we used an 18-gauge needle to perform a single pass through the cecum. After removing the needle, we squeezed a tiny bit of fetal material to verify the holes. We inserted the cecum into the abdomen and used a 3.0 silk suture to repair the opening. We administered 50 mg/kg saline subcutaneously for resuscitation. After the experiment, we returned the rats to their cages. The animals had free access to food and water. About 16 hours are required to create a mild-grade CLP-induced sepsis model by medium ligation with an 18-gauge needle (38). Thus, we sacrificed the rats following the 16^th^ hour through high-dose anesthesia to collect the renal and lung tissues and blood samples.

Group III (CLP+COST 5 mg/kg, n=10): The same procedures were performed with group II, but 5 mg/kg COST was administered IP 30 min before the CLP model.

Group IV (CLP+COST 10 mg/kg, n=10): 10 mg/kg COST was applied IP before the CLP model.

***Biochemical analysis***

Lung and renal tissue samples were ground with liquid nitrogen for homogenization. Next, centrifugation was performed for 30 min at 5000 rpm. SOD, MDA, and GSH levels were measured by calorimetric methods as described in previous studies (39-41). Serum NF-κB, TNF-α, IL-6 and IL-1β (Catalog No: BLS-1693Ra, BLS-1396Ra, BLS-1158Ra, BLS-1272Ra, respectively, Bostonchem, USA) levels were assessed through an ELISA reader (ELISA, BioTEK PowerWave XS Winooski, UK). 

***Histopathological procedures***

The samples including kidney and lung tissues were collected and fixed in 10% buffered formalin, dehydrated in graded alcohol solution, cleared with xylene, and embedded in paraffin. Hematoxylin and eosin (H&E) were used to stain tissue sections of 5 mm thickness. After that, the sections were examined using a light microscope (Olympus BX51, Tokyo, Japan). 

***Immunohistochemical procedures***

The procedure and protocol for immunohistochemical staining were based on our recent study (33). In brief, rehydrated paraffin sections were quenched with 3% H_2_O_2_ for 10 min before being incubated with antigen retrieval solution. Sections were then incubated with a primary antibody specifically against 8-OHdG (sc-66036, 1:100, Santa Cruz Biotechnology) for 60 min at room temperature. The secondary antibody (Mouse and Rabbit Specific HRP/DAB IHC Detection Kit - Micro-polymer, ab236466, Abcam) was then added and incubated for 10 min before being DAB stained, dehydrated after hematoxylin counterstaining, cleared in xylene, and mounted. 8-OHdG immunopositivity was scored as follows: none (0), mild (1), moderate (2), and intense (3).

***Gene expression analysis***

Lung (30 mg) and kidney (30 mg) tissues were treated using RNA stabilization reagent. The tissues were then frozen with liquid nitrogen and homogenized with Tissue Lyser II. Total RNA was extracted according to the manufacturer’s instructions. The RNA samples were reverse-transcribed into complementary DNA using a high-capacity cDNA reverse transcription kit. The Epoch Spectrophotometer System and Take3 Plate were used to determine and quantify cDNA concentrations (42). Materials used for the processes are represented in Table 1. 

The Step One Plus Real-Time PCR System technology (Applied Biosystems) was used to analyze the expression of TNF-α, IL1-β, IL-6, and NF-κB. Endogenous controls were carried out through beta-actin. All quantifications of gene expression procedures were carried out for each group in triplicate determinations in a 96-well optical PCR plate. The 2^−ΔΔCt^ method (43) represented all data as fold changes in expression compared to the SHAM group.

***Statistical analysis***

We used SPSS 20.0 software (IBM Corp, Armonk, NY, USA) for statistical analysis. The results were shown as mean and standard deviation. To compare the groups, one-way ANOVA and Duncan’s multiple comparison tests were utilized. Statistical significance was defined as a *P*-value of 0.05. In the Duncan test, means with the same letter in the same column are not statistically different. 

For the immunohistochemical examination, data are presented as mean ± standard error (SE). GraphPad Prism 8.0.1 software was used in the statistical analysis. Kruskal Wallis followed by the Mann-Whitney U test was performed to compare the differences among groups. *P*<0.05 was regarded as statistically significant.

## Results

***COST alleviates oxidative stress in septic lung and kidney tissues***

**Figure 5:**
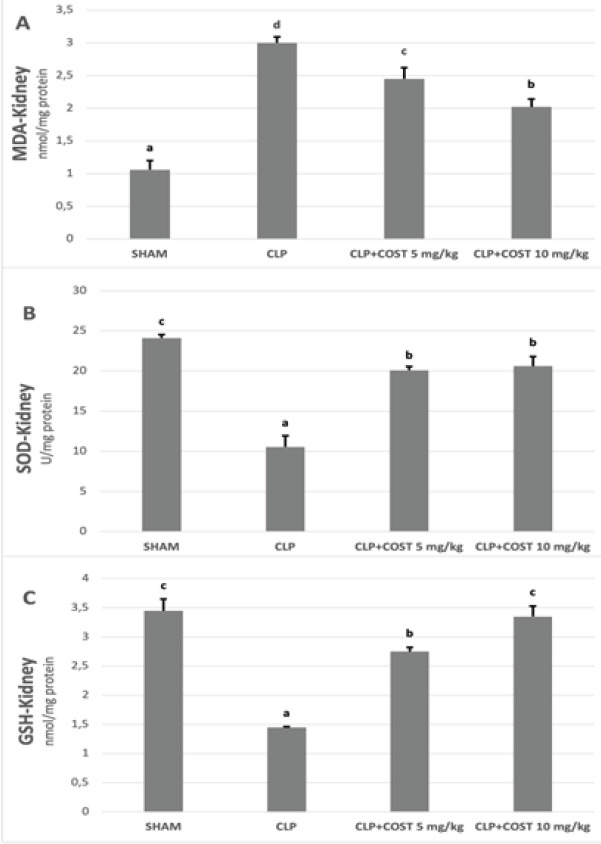
Kidney GSH, SOD, and MDA levels of the experimental groups in the cecal ligation and puncture rat model

In the kidney tissue (Figure 5), COST significantly reduced the MDA levels and elevated the SOD and GSH activity, compared to the CLP group (*P*<0.05). High-dose COST administration (10 mg/kg) was more efficient than the low dose (5 mg/kg) statistically (*P*<0.05) for MDA and GSH values. It was also higher, but not meaningful for SOD activity (*P*>0.05).

**Figure 6:**
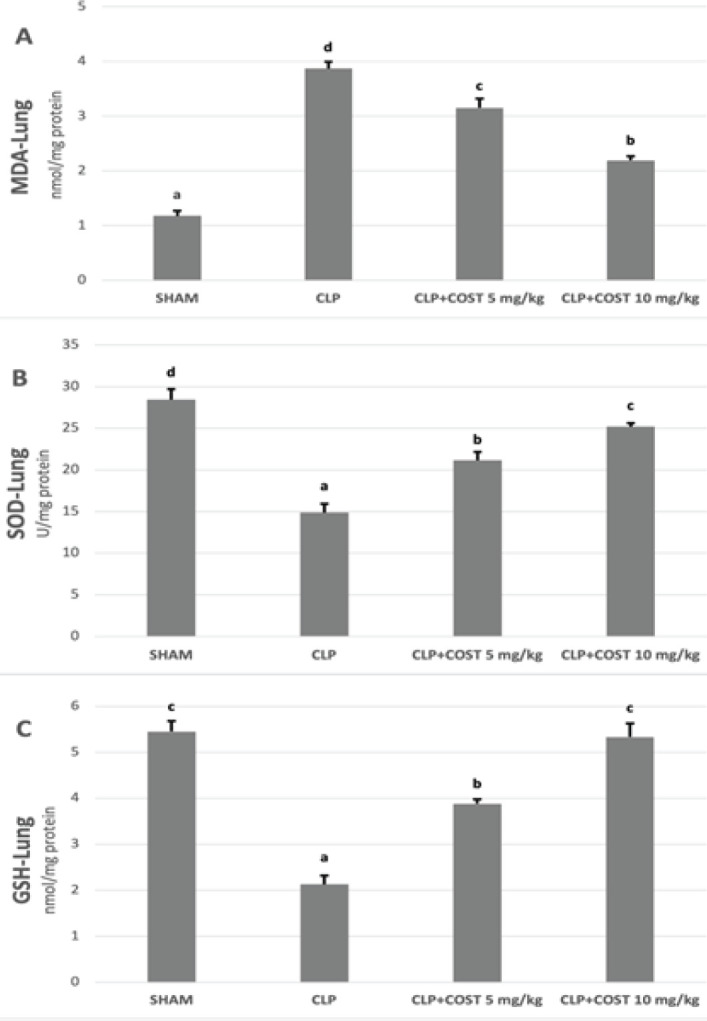
Lung GSH, SOD, and MDA levels of the experimental groups in the cecal ligation and puncture rat model

In the lung tissue (Figure 6), MDA levels decreased and GSH and SOD activity elevated in the COST groups compared to the CLP group (*P*<0.05). High dose COST group performed a better anti-oxidant activity than the low-dose COST group in terms of MDA, GSH, and SOD levels (*P*<0.05). 

***COST performs anti-inflammatory activity through attenuating proinflammatory cytokine production***

**Figure 7:**
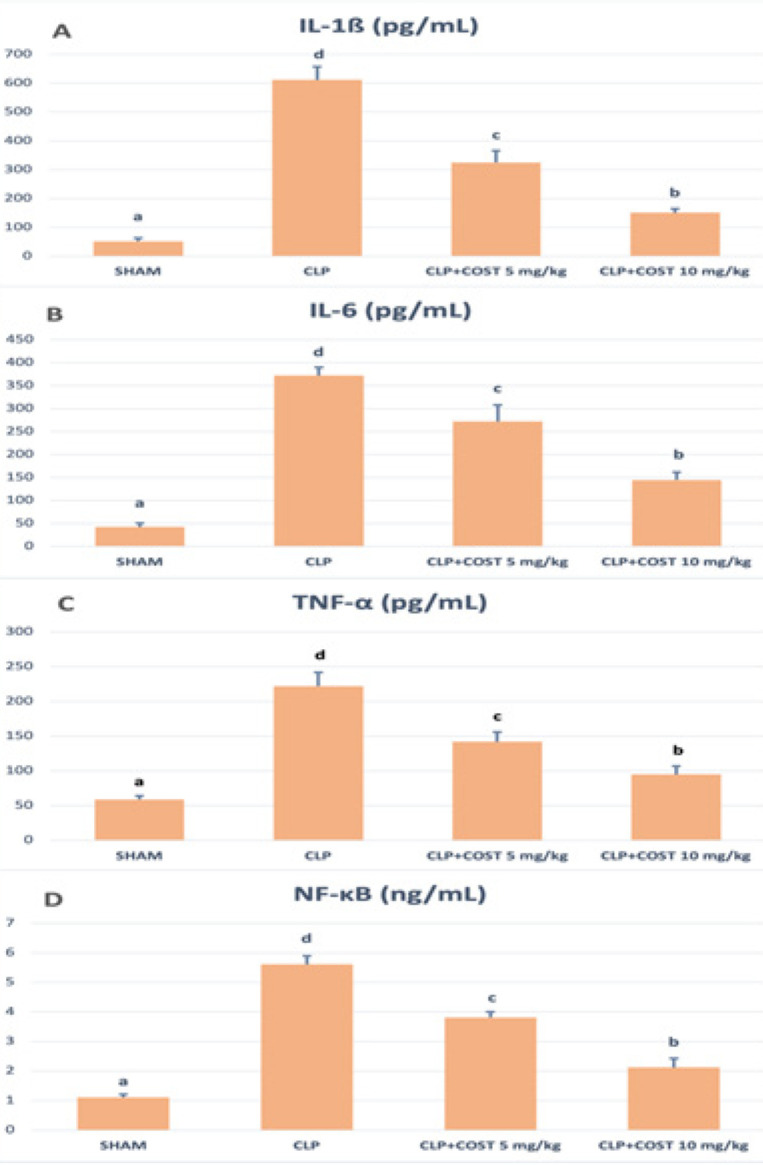
TNF-α, IL1-β, IL-6, and NF-κB levels of the experimental groups in the cecal ligation and puncture rat model

Figure 7 represents the serum cytokine levels of the experimental groups. COST lowered the TNF-α, IL1-β, IL-6, and NF-κB levels significantly compared to the CLP group (*P*<0.05). When the COST groups were compared to each other, high dose COST administration left standing the low dose (*P*<0.05). 

**Figure 8:**
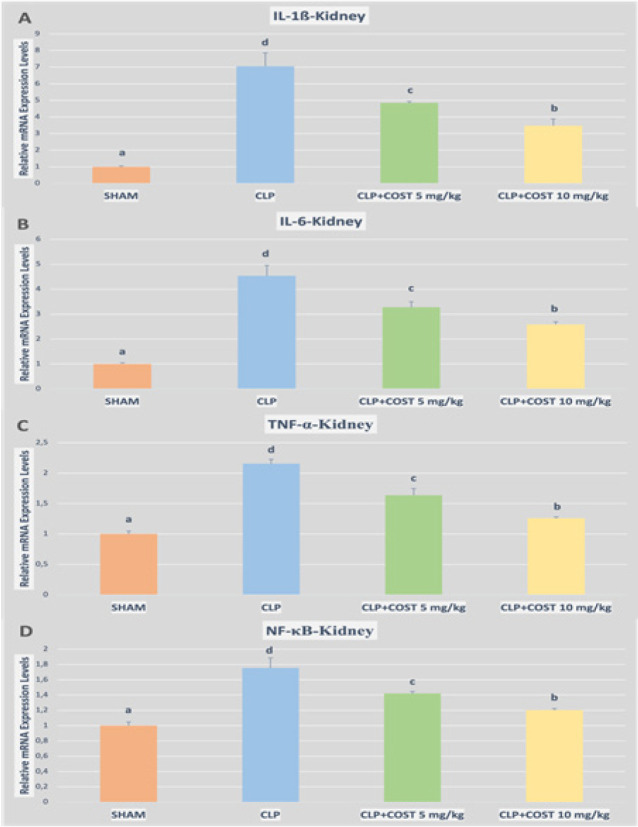
Relative mRNA expression levels of IL1-β, IL-6, TNF-α, and NFκB of the kidney tissues in the cecal ligation and puncture rat model

**Figure 9:**
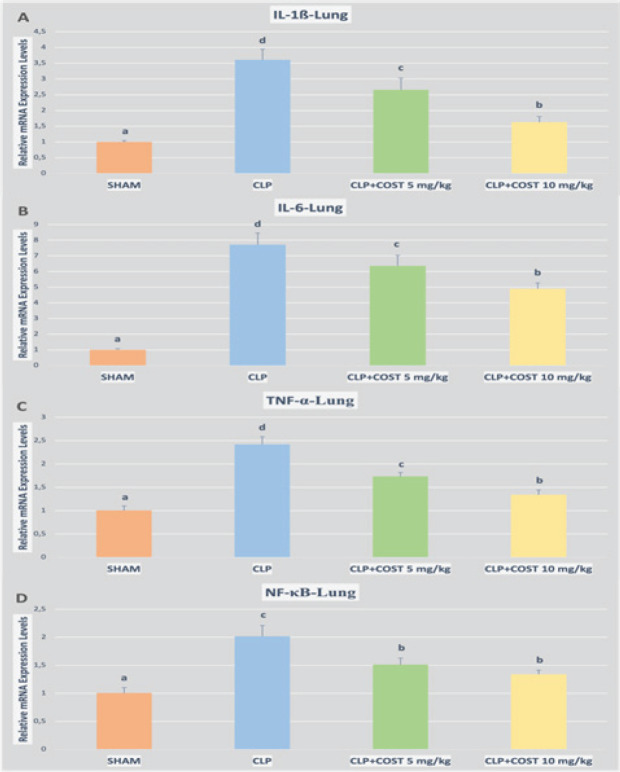
Relative mRNA expression levels of IL1-β, IL-6, TNF-α, and NFκB of the lung tissues in the cecal ligation and puncture rat model

Besides, the lung and kidney tissue cytokine expression levels were in accordance with the ELISA results. COST diminished TNF-α, IL1-β, IL-6, and NF-κB expression in both kidney and lung tissue samples (*P*<0.05, Figures 8 and 9, respectively). The high-dose COST group was more effective than the low-dose group for alleviating both kidney and lung cytokine expression, while the difference did not make sense for only NF-κB expression in the lung tissue (*P*>0.05).

***Effects of COST on histopathology of the kidney and lung tissues after the CLP procedure***

**Figure 10:**
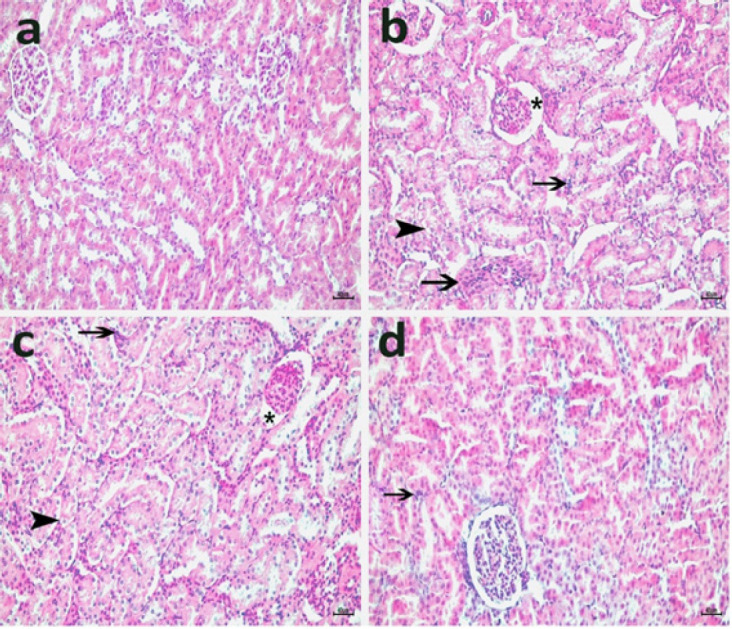
Protective effect of COST against CLP-induced kidney injury in the cecal ligation and puncture rat model

Figure 10 demonstrates the kidney tissue histopathological findings. As seen in Figure 10a, the glomerulus structure was typical architecture, and the tubules were firmly packed in the SHAM group. In the CLP group, signs of widened Bowman’s capsule were observed (Figure 10b). Additionally, mononuclear cell infiltration in interstitial areas and focal tubular ballooning degeneration were signs of tubular injury. Furthermore, the CLP+COST 5mg/kg group showed moderately enlarged Bowman’s capsule, cytoplasmic vacuolations, and inflammatory cellular infiltrates (Figure 10c). The CLP+COST 10 mg group had almost normal renal glomeruli appearance, moderate tubular cytoplasmic vacuolation, and inflammatory cellular infiltrates (Figure 10d).

**Figure 11:**
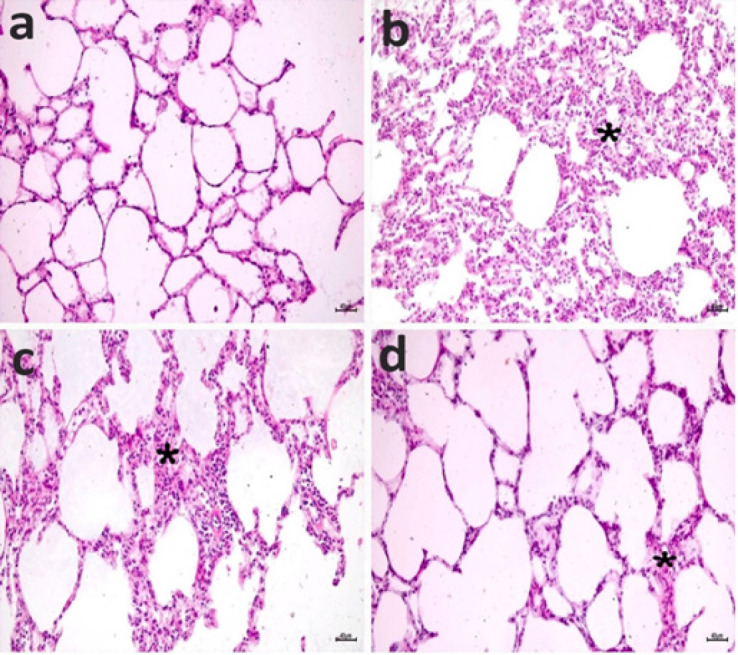
**Protective effect of COST against CLP-induced lung injury in the cecal ligation and puncture rat model**SHAM group (a), CLP group (b), CLP+COST 5 mg/kg group (c), CLP+COST 10 mg/kg group (d). Thickening due to inflammatory infiltrate (asterisk). H&Ex200.

Histopathological findings of the lung tissue are shown in Figure 11. The alveoli structure in the SHAM group was regular (Figure 11a). Edema and inflammatory infiltration (alveolar macrophages and lymphocytes) thickened the alveolar membranes in the CLP group (Figure 11b). After the administration of 5 mg/kg COST, moderate inflammatory infiltrates were seen in alveolar membranes (Figure 11c). CLP+10 mg COST group showed nearly normal alveoli structure and mild inflammatory infiltrates in alveolar membranes (Figure 11d).

***COST ameliorates CLP-Induced 8-OHdG immunopositivity***

**Figure 12:**
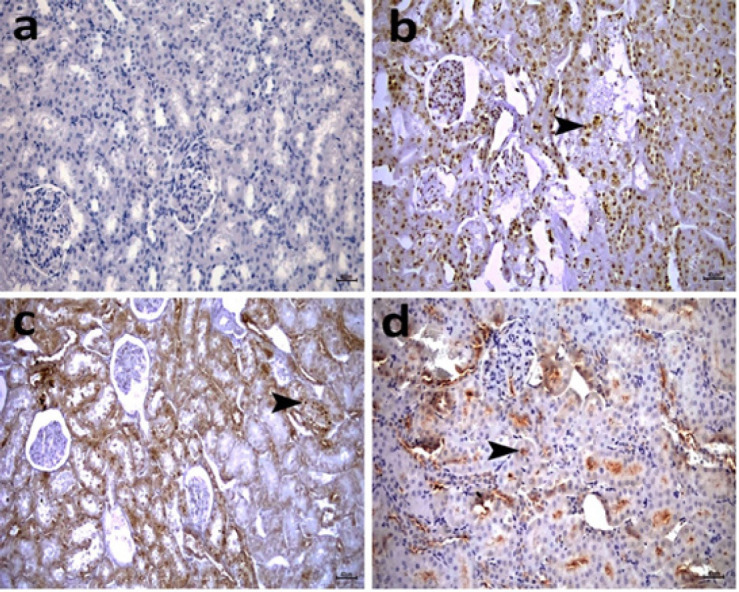
Representative photomicrographs of rat renal tissues immunopositive for 8-OHdG from the sham group showing no immunopositivity in the renal tubules (a), from the CLP group (b) with intense immunopositivity in the renal tubules (arrowhead), from the CLP+ COST 5 mg/kg (c) treated group showing moderate immunopositivity for 8-OHdG in the renal tubules (arrowhead), and from the CLP+ COST 10 mg/kg (d) treated group showing mild immunopositivity for 8-OHdG in the renal tubules (arrowhead). IHCx200

**Figure 13:**
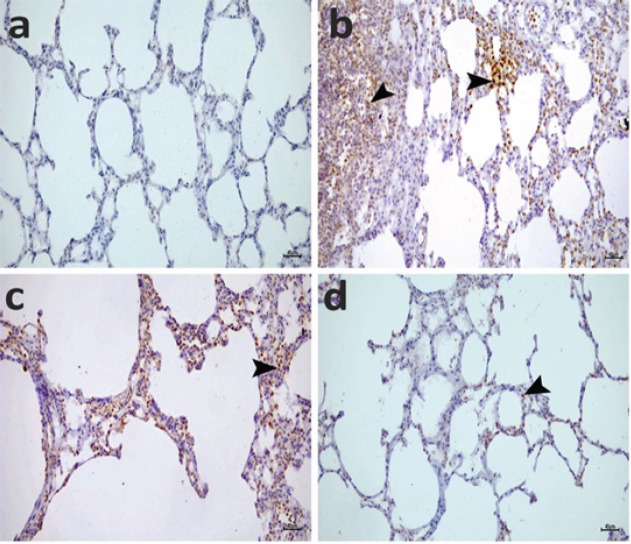
Representative photomicrographs of rat lung tissues immunopositive for 8-OHdG from the SHAM group (a) showing no expression in the cells of the alveoli, from the CLP group (b) with intense immunopositivity in the cells of the interalveolar areas (arrowheads), from the CLP+COST 5 mg/kg (c) treated group showing moderate immunopositivity for 8-OHdG in the cells of the alveoli (arrowhead), and from the CLP+COST 10 mg/kg (d) treated group showing mild immunopositivity for 8-OHdG in the cells of the alveoli (arrowhead). IHCx200

As shown in Figures 12a and 13a, the lung and kidney sections represented no immunopositivity for 8-OHdG in the SHAM group (Table 2, *P*>0.05), but intense positive immunopositivity was detected in the CLP group (Figures 12b and 13b; Table 1, *P*<0.05). CLP+ COST 5 mg showed moderate 8-OHdG immunopositivity (Figures 12c and 13c; Table 2, *P*<0.05). Administration of 10 mg COST attenuated the rise of immunopositivity in lung and kidney sections after CLP induction. The renal tubules and cells lining the alveoli showed a mild 8-OHdG immunopositivity in the CLP+COST 10 mg group (Figures 12d and 13d; Table 2, *P*<0.05).

COST: Costunolide

CLP: Cecal ligation and puncture

CLP: Cecal ligation and puncture, COST: Costunolide, IP: Intraperitoneal, DMSO: Dimethyl sulfoxide, PBS: Phosphate buffer solution

**Table 1:** Gene express analysis materials for the Step One Plus Real-Time PCR system

**cDNA synthesis kit** cDNA Synthesis Kit with Rnase Inh. (High Capacity)	A.B.T. Laboratory Industry, Turkey	**Catalog No** C03-01-20
**Mastermix components** 2x Amplifyme Probe Mix PCR grade water 50x High ROX Solution	Blirt AMPLIFYME Probe Universal Mix	**Lot No** ACX756077 ACW836075 ADS566275
**Primers** Rat-TNF -F Rat-TNF -R Rat-IL6-F Rat-IL6-R Rat-IL1- -F Rat-IL1- -R Rat- NF- B-F Rat- NF- B-R Rat-ACTB-F Rat-ACTB-R Rat-TNF Probe Rat-IL6 Probe Rat-IL1 Probe Rat- NF- B Probe Rat-ACTB Probe	TIB Molbiol	**Catalog No** 2203398 2203399 2203400 2203401 2203402 2203403 2203404 2203405 2203406 2203407 2203408 2203409 2203410 2203411 2203412

Statistically, *P*<0.05 value was considered significant. Different letters (a, b, c, and d) indicate the statistical differences. There are no statistically significant differences between groups that share a common letter

COST: Costunolide, CLP: Cecal ligation and puncture, MDA: Malondialdehyde, SOD: Superoxide dismutase, GSH: Glutathione

Statistically, *P*<0.05 was considered significant. Different letters (a, b, c, and d) indicate the statistical differences. There are no statistically significant differences between groups that share a common letter

COST: Costunolide, CLP: Cecal ligation and puncture, MDA: Malondialdehyde, SOD: Superoxide dismutase, GSH: Glutathione

Statistically, *P*<0.05 value was considered significant. Different letters (a, b, c, and d) indicate the statistical differences. There are no statistically significant differences between groups that share a common letter. IL-1β Interleukin-1beta IL-6: interleukin-6, TNF-α: Tumor necrosis factor-alpha, NF-κB: Nuclear factor kappa-B

The expression of mRNAs was detected using quantitative Real-Time PCR analysis. β-actin was used as the reference gene. Statistically, *P*<0.05 value was considered significant. Different letters (a, b, c, and d) indicate the statistical differences. There are no statistically significant differences between groups that share a common letter

IL-1β: Interleukin-1beta, IL-6: interleukin-6, TNF-α: Tumor necrosis factor-alpha, NF-κB: Nuclear factor kappa-B

The expression of mRNAs was detected using quantitative Real-Time PCR analysis. β-actin was used as the reference gene. Statistically, *P*<0.05 was considered significant. Different letters (a, b, c, and d) indicate the statistical differences. There are no statistically significant differences between groups that share a common letter

IL-1β: Interleukin-1beta IL-6: interleukin-6, TNF-α: Tumor necrosis factor-alpha, NF-κB: Nuclear factor kappa-B

SHAM group (a), CLP group (b), CLP+COST 5 mg/kg group (c), CLP+ COST 10 mg/kg group (d). Enlarged Bowman's spaces (asterisks), cytoplasmic vacuolations (arrowheads), and inflammatory cellular infiltrates (arrow). H&Ex200.

COST: Costunolide, CLP: Cecal ligation and puncture

COST: Costunolide, CLP: Cecal ligation and puncture

COST: Costunolide, CLP: Cecal ligation and puncture

**Table 2:** The effect of COST treatment on 8-OHdG immunopositivity of lung and kidney tissues in CLP rat experimental groups

Groups/Antibody	SHAM	CLP	CLP+COST 5 mg	CLP+COST 10 mg
Lung/8-OHdG	0.30±0.15	2.70±0.21^*^	2.10±0.17^*#^	0.70±0.15^#+^
Kidney/8-OHdG	0.10±0.10	2.90±0.10^*^	2.00±0.14^*#^	0.60±0.16^#+^

Data are expressed as mean ± SE (n = 10)

*significant difference with the SHAM group

#significant difference with the CLP group

+significant difference with CLP+5 mg COST group

COST: Costunolide, CLP: Cecal ligation and puncture

COST: Costunolide, CLP: Cecal ligation and puncture

## Discussion

This study investigates the potential protective effects of COST against the sepsis model caused by CLP in rats by assessing oxidative stress and inflammation. The CLP-induced sepsis model was validated through overexpression of TNF-α, IL1-β, IL-6, and NF-kB, thereby demonstrating the occurrence of a systemic inflammatory response. Furthermore, the generation of the identical parameters was corroborated by the ELISA findings. The presence of 8-OHdG immunopositivity and the measurement of SOD, GSH, and MDA levels in lung and renal tissue samples provided evidence of oxidative stress associated with sepsis. The histological examination offered additional support to the conclusions. COST injection effectively mitigated the situation and averted sepsis generated by CLP. The results demonstrated the preventive effects of COST.

Sepsis is a critical medical disorder that poses a significant risk to life, characterized by a close association with systemic inflammation and the occurrence of multiple organ failure (44). The primary cause of organ damage in sepsis is inflammation (45). The kidneys and lungs are among the organs commonly affected by sepsis (9). During inflammation, there is excessive production of several inflammatory mediators such as TNF-α, IL1-β, IL-6, and NF-κB, among others (46). Multiple proinflammatory genes, such as TNF-α, IL1-β, and IL-6, depend on the transcription factor NF-κB for their production (47). 

COST suppresses the NF-kB signaling pathway, implying that it has anti-inflammatory properties (27). Prior research demonstrated that COST inhibited chronic inflammation induced by NF-κB and Wnt/β-catenin signaling pathways (48). The study conducted by Mao *et al*. demonstrated that COST mitigated the detrimental effects of alcohol-induced liver injury by modulating the LPS/TLR4/NF-κB signaling pathway (49). The administration of COST resulted in decreased phosphorylation of NF-κB p65 in mice colon tissues induced by dextran sulfate sodium (DSS) (50). Inhibition of NF-κB-mediated inflammation by COST resulted in the alleviation of atherosclerosis in mice (51). COST also reduced NF-B activity in LPS-stimulated RAW264.7 cells in a different study (52). Pitchai et al. observed a reduction in the overexpression of NF-κB subunits in breast cancer cells due to treatment with COST (53). NF-κB activation was suppressed by COST administration in acute liver injury (54), pleurisy (55), and acute gastric ulcer (56) mouse models. Our results are consistent with the literature, and COST inhibited NF-κB production in our CLP-induced sepsis rat model. 

Sepsis induces an up-regulation in the production of proinflammatory cytokines, including IL1-β, IL-6, and TNF-α (57). The administration of COST resulted in the reduction of TNF-α, IL1-β, and IL-6 levels in an obesity cardiomyopathy mouse model (58). COST decreased the expression of IL1- β, IL-6, and TNF-α in a murine model of acute ulcerative colitis, according to another study (50). COST effectively mitigated the expression of hepatic proinflammatory cytokines, including  IL-1β, IL-6, and TNF-α, in mice fed the DCC diet (59). The treatment of COST resulted in a reduction in the level of TNF-α in a rat model of cerebral ischemia (60). Treatment with COST dramatically lowered IL-6 and TNF-α levels and improved lipoteichoic acid (LTA)-induced inflammatory response in an acute lung injury mouse model (61). Wang *et al*. reported that COST treatment decreased the expression of TNF- and IL1- in a murine model of acute liver injury (54). COST prevented TNF-α production in an experimental pleurisy study (55) and an ethanol-induced gastric ulcer rat model (56). In our previous study, we created a renal ischemia-reperfusion rat model and first experienced COST and alleviated the inflammatory process by diminishing IL1-β, IL-6, and TNF-α levels (33). Here, we managed to decrease IL1-β, IL-6, and TNF-α levels with COST administration. Our data was in accordance with the literature, mentioning COST’s anti-inflammatory properties through affecting pro-inflammatory genes. 

In sepsis models, there is a drop in the levels of SOD and GSH while the levels of MDA increase. These changes can be attributed to the presence of oxidative stress (62, 63). In an experimental rat model, the administration of COST increased SOD activity in response to cerebral ischemia (60). The enzymatic activities of GSH and SOD were enhanced following treatment with COST in a rat model of diabetes induced by streptozotocin (STZ) (64). In a model of acute gastric rat ulcer, treatment with COST resulted in increased SOD activity and regression of MDA levels, indicating enhanced antioxidant activity (56). Our previous research revealed that COST induced SOD and GSH activity and decreased MDA value by showing an antioxidant effect in a renal ischemia-reperfusion rat model (33). In the current study, we obtained compatible results with the literature and found out the antioxidant capacity of COST in sepsis.

8-OHdG is a recognized biomarker of oxidative DNA damage and exhibits elevated levels in instances of organ harm associated with sepsis (65). In our previous research, COST diminished 8-OHdG expression in lung and kidney tissues in an ischemia-reperfusion rat model and prevented oxidative DNA damage (33). COST inhibited 8-OHdG expression in lung and kidney tissues in our study, indicating the preventive effect of COST against oxidative DNA damage. 

The kidneys and lungs are susceptible to harm associated with sepsis (63, 66). Sepsis-induced organ damage is closely linked to the occurrence of systemic inflammation (67). Although we examined the inflammatory mediator expression, we also histologically investigated lung and kidney tissue samples, supporting our findings.

The present study demonstrated that the administration of COST significantly ameliorated the detrimental effects of CLP-induced sepsis by enhancing inflammatory indices and oxidative stress markers. COST exhibited both anti-inflammatory properties against systemic inflammation and protective effects on the lungs and kidneys, which are two vital organs susceptible to septic damage. 

## Conclusion

Our study showed that COST performed anti-inflammatory and antioxidant activity by alleviating CLP-induced sepsis injury in rats. COST mitigated systemic inflammation by suppressing proinflammatory cytokine production and attenuated oxidative stress via improving oxidant and antioxidant parameters. Histological examination supported our findings. 

The current findings highlight the potent benefits of COST as an appropriate candidate for sepsis and sepsis-related organ failure therapies. Our research was the first experiment on the effects of COST against sepsis, and we suggest that the data is worth evaluating for further research. 

## Authors’ Contributions

MC G, E E, A T, Ö Ç, and D Ç designed the experiments; MC G, E E, A T, Ö Ç, and D Ç performed experiments and collected data; MC G, Y B, S Ç, and EE Y performed analysis and interpretation of the results; MC G, E A, A T, Ö Ç, D Ç, S Ç, Y B, and EEY discussed the results and strategy; MC G supervised, directed, and managed the study; MC G, E A, A T, Ö Ç, D Ç, S Ç, EE Y, and YB approved the final version to be published.

## Limitations

Despite the encouraging results, our study has limitations. We limited the outcome to female Wistar Albino rats. In addition, even though the CLP-induced sepsis model is safe and widespread due to its similarity to human sepsis, we did not experience other models. Because this is the first COST study on the sepsis model, we focused on inflammation and oxidative stress markers in the short term. However, our findings paved the way for *in vivo *and* in silico* studies to improve and understand COST for sepsis treatment.

## Conflicts of Interest

The authors declare that they have no conflicts of interest.

## References

[1] Abdelnaser M, Alaaeldin R, Attya ME, Fathy M (2023). Hepatoprotective potential of gabapentin in cecal ligation and puncture-induced sepsis; targeting oxidative stress, apoptosis, and NF-kB/MAPK signaling pathways. Life Sci.

[2] Hotchkiss RS, Moldawer LL, Opal SM, Reinhart K, Turnbull IR, Vincent J-L (2016). Sepsis and septic shock. Nat Rev Dis Primers.

[3] Cecconi M, Evans L, Levy M, Rhodes A (2018). Sepsis and septic shock. Lancet.

[4] Font MD, Thyagarajan B, Khanna AK (2020). Sepsis and septic shock – basics of diagnosis, pathophysiology and clinical decision making. Med Clin North Am.

[5] Stanski NL, Wong HR (2020). Prognostic and predictive enrichment in sepsis. Nat Rev Nephrol.

[6] Rudd KE, Johnson SC, Agesa KM, Shackelford KA, Tsoi D, Kievlan DR (2020). Global, regional, and national sepsis incidence and mortality, 1990–2017: Analysis for the global burden of disease study. Lancet.

[7] Chen L, Huang Q, Zhao T, Sui L, Wang S, Xiao Z (2021). Nanotherapies for sepsis by regulating inflammatory signals and reactive oxygen and nitrogen species: New insight for treating COVID-19. Redox Biol.

[8] Fleischmann-Struzek C, Mellhammar L, Rose N, Cassini A, Rudd KE, Schlattmann P (2020). Incidence and mortality of hospital- and ICU-treated sepsis: Results from an updated and expanded systematic review and meta-analysis. Intensive Care Med.

[9] Lan KC, Chao SC, Wu HY, Chiang CL, Wang CC, Liu SH (2017). Salidroside ameliorates sepsis-induced acute lung injury and mortality via downregulating NF-κB and HMGB1 pathways through the upregulation of SIRT1. Sci Rep.

[10] Lu J, Li Y, Gong S, Wang J, Lu X, Jin Q (2022). Ciclopirox targets cellular bioenergetics and activates ER stress to induce apoptosis in non-small cell lung cancer cells. Cell Commun Signal.

[11] Burton GJ, Jauniaux E (2011). Oxidative stress. Best Pract Res Clin Obstet Gynaecol.

[12] Yao X, Carlson D, Sun Y, Ma L, Wolf SE, Minei JP (2015). Mitochondrial ROS induces cardiac inflammation via a pathway through mtDNA damage in a pneumonia-related sepsis model. PLoS ONE.

[13] Huang HC, Hsiao TS, Liao MH, Tsao CM, Shih CC, Wu CC (2020). Low-dose hydralazine improves endotoxin-induced coagulopathy and multiple organ dysfunction via its anti-inflammatory and anti-oxidative/nitrosative properties. Eur J Pharmacol.

[14] Ji M-h, Xia D-g, Zhu L-y, Zhu X, Zhou X-y, Xia J-y (2018). Short- and long-term protective effects of melatonin in a mouse model of sepsis-associated encephalopathy. Inflammation.

[15] Kim MH, Kim JN, Han SN, Kim HK (2015). Ursolic acid isolated from guava leaves inhibits inflammatory mediators and reactive oxygen species in LPS-stimulated macrophages. Immunopharmacol Immunotoxicol.

[16] Wu D, Liu B, Yin J, Xu T, Zhao S, Xu Q (2017). Detection of 8-hydroxydeoxyguanosine (8-OHdG) as a biomarker of oxidative damage in peripheral leukocyte DNA by UHPLC-MS/MS. J Chromatogr B Analyt Technol Biomed Life Sci.

[17] Aboyoussef AM, Mohammad MK, Abo-Saif AA, Messiha BAS (2021). Granisetron attenuates liver injury and inflammation in a rat model of cecal ligation and puncture-induced sepsis. J Pharmacol Sci.

[18] Chen K, Wu L, Liu Q, Tan F, Wang L, Zhao D (2023). Glutathione improves testicular spermatogenesis through inhibiting oxidative stress, mitochondrial damage, and apoptosis induced by copper deposition in mice with Wilson disease. Biomed Pharmacother.

[19] Bui QTN, Ki J-S (2023). Two novel superoxide dismutase genes (CuZnSOD and MnSOD) in the toxic marine dinoflagellate alexandrium pacificum and their differential responses to metal stressors. Chemosphere.

[20] Huang C, Tong L, Lu X, Wang J, Yao W, Jiang B (2015). Methylene blue attenuates iNOS induction through suppression of transcriptional factor binding amid iNOS mRNA transcription. J Cell Biochem.

[21] Venet F, Monneret G (2018). Advances in the understanding and treatment of sepsis-induced immunosuppression. Nat Rev Nephrol.

[22] Yin G, Liu J, Zhang Y, Yang G (2022). Effect of 5-aminolevulinic acid photodynamic therapy on the expression of toll-like receptor 4 and nuclear factor kappa B in condyloma acuminatum keratinocytes. Photodiagnosis Photodyn Ther.

[23] He X, Liu W, Shi M, Yang Z, Zhang X, Gong P (2017). Docosahexaenoic acid attenuates LPS-stimulated inflammatory response by regulating the PPARγ/NF-κB pathways in primary bovine mammary epithelial cells. Res Vet Sci.

[24] Seymour CW, Liu VX, Iwashyna TJ, Brunkhorst FM, Rea TD, Scherag A (2016). Assessment of clinical criteria for sepsis: For the third international consensus definitions for sepsis and septic shock (sepsis-3). JAMA.

[25] Chaudhry H, Zhou J, Zhong Y, Ali MM, McGuire F, Nagarkatti PS (2013). Role of cytokines as a double-edged sword in sepsis. In Vivo.

[26] Lv Q, Xing Y, Dong D, Hu Y, Chen Q, Zhai L (2021). Costunolide ameliorates colitis via specific inhibition of HIF1α/glycolysis-mediated Th17 differentiation. Int Immunopharmacol.

[27] Kim DY, Choi BY (2019). Costunolide—a bioactive sesquiterpene lactone with diverse therapeutic potential. Intl J Mol Sci.

[28] Alotaibi AA, Bepari A, Assiri RA, Niazi SK, Nayaka S, Rudrappa M (2021). Saussurea lappa exhibits anti-oncogenic effect in hepatocellular carcinoma, HepG2 cancer cell line by Bcl-2 mediated apoptotic pathway and mitochondrial cytochrome C release. Curr Mol Biol.

[29] Schabbauer G (2012). Polymicrobial sepsis models: CLP versus CASP. Drug Discov Today.

[30] Brooks HF, Moss RF, Davies NA, Jalan R, Davies DC (2014). Caecal ligation and puncture induced sepsis in the rat results in increased brain water content and perimicrovessel oedema. Metab Brain Dis.

[31] Stortz JA, Raymond SL, Mira JC, Moldawer LL, Mohr AM, Efron PA (2017). Murine Models of Sepsis and Trauma: Can We Bridge the Gap?. Ilar j.

[32] Huang H, Park S, Zhang H, Park S, Kwon W, Kim E (2021). Targeting AKT with costunolide suppresses the growth of colorectal cancer cells and induces apoptosis in vitro and in vivo. J Exp Clin Cancer Res.

[33] Güler MC, Akpinar E, Tanyeli A, Çomakli S, Bayir Y (2023). Costunolide prevents renal ischemia-reperfusion injury in rats by reducing autophagy, apoptosis, inflammation, and DNA damage. Iran J Basic Med Sci.

[34] Bayraktutan Z, Dincer B, Keskin H, Kose D, Bilen A, Toktay E (2022). Roflumilast as a potential therapeutic agent for cecal ligation and puncture-induced septic lung injury. J Invest Surg.

[35] Güler MC, Tanyeli A, Eraslan E, Çomaklı S, Bayır Y (2022). Cecal ligation and puncture-induced sepsis model in rats. J Lab Animal Sci Pract.

[36] Song L, Zou Y, Cao Z (2018). Comparison of two different models of sepsis induced by cecal ligation and puncture in rats. J Surg Res.

[37] Ruiz S, Vardon-Bounes F, Merlet-Dupuy V, Conil JM, Buléon M, Fourcade O (2016). Sepsis modeling in mice: Ligation length is a major severity factor in cecal ligation and puncture. Intensive Care Med Exp.

[38] Hubbard WJ, Choudhry M, Schwacha MG, Kerby JD, Rue LW 3rd, Bland KI (2005). Cecal ligation and puncture. Shock.

[39] Hu ML (1994). Measurement of protein thiol groups and glutathione in plasma. Methods Enzymol.

[40] Ohkawa H, Ohishi N, Yagi K (1979). Assay for lipid peroxides in animal tissues by thiobarbituric acid reaction. Anal Biochem.

[41] Sun Y, Oberley LW, Li Y (1988). A simple method for clinical assay of superoxide dismutase. Clin Chem.

[42] Un H, Ugan RA, Kose D, Yayla M, Tastan TB, Bayir Y (2022). A new approach to sepsis treatment by rasagiline: A molecular, biochemical and histopathological study. Mol Biol Rep.

[43] Livak KJ, Schmittgen TD (2001). Analysis of relative gene expression data using real-time quantitative PCR and the 2(-Delta Delta C(T)) Method. Methods.

[44] Prescott HC, Angus DC (2018). Enhancing recovery from sepsis: A review. JAMA.

[45] Gao J, Zhao F, Yi S, Li S, Zhu A, Tang Y (2022). Protective role of crocin against sepsis-induced injury in the liver, kidney and lungs via inhibition of p38 MAPK/NF-κB and Bax/Bcl-2 signalling pathways. Pharm Biol.

[46] Khajevand-Khazaei M-R, Mohseni-Moghaddam P, Hosseini M, Gholami L, Baluchnejadmojarad T, Roghani M (2018). Rutin, a quercetin glycoside, alleviates acute endotoxemic kidney injury in C57BL/6 mice via suppression of inflammation and up-regulation of antioxidants and SIRT1. Eur J Pharmacol.

[47] Atreya I, Atreya R, Neurath MF (2008). NF-κB in inflammatory bowel disease. J Intern Med.

[48] He Y, Moqbel SAA, Xu L, Ran J, Ma C, Xu K (2019). Costunolide inhibits matrix metalloproteinases expression and osteoarthritis via the NFκB and Wnt/βcatenin signaling pathways. Mol Med Rep.

[49] Mao J, Zhan H, Meng F, Wang G, Huang D, Liao Z (2022). Costunolide protects against alcohol-induced liver injury by regulating gut microbiota, oxidative stress and attenuating inflammation in vivo and in vitro. Phytother Res.

[50] Xie F, Zhang H, Zheng C, Shen X-f (2020). Costunolide improved dextran sulfate sodium-induced acute ulcerative colitis in mice through NF-κB, STAT1/3, and Akt signaling pathways. Int Immunopharmacol.

[51] Huang Z-q, Luo W, Li W-x, Chen P, Wang Z, Chen R-j (2023). Costunolide alleviates atherosclerosis in high-fat diet-fed ApoE−/− mice through covalently binding to IKKβ and inhibiting NF-κB-mediated inflammation. Acta Pharmacologica Sinica.

[52] Kang JS, Yoon YD, Lee KH, Park S-K, Kim HM (2004). Costunolide inhibits interleukin-1β expression by down-regulation of AP-1 and MAPK activity in LPS-stimulated RAW 264 7 cells. Biochem Biophys Res Commun.

[53] Pitchai D, Roy A, Banu S (2014). In vitro and in silico evaluation of NF-κB targeted costunolide action on estrogen receptor-negative breast cancer cells—a comparison with normal breast cells. Phytother Res.

[54] Wang Y, Zhang X, Zhao L, Shi M, Wei Z, Yang Z (2017). Costunolide protects lipopolysaccharide/d-galactosamine–induced acute liver injury in mice by inhibiting NF-κB signaling pathway. J Surg Res.

[55] Butturini E, Di Paola R, Suzuki H, Paterniti I, Ahmad A, Mariotto S (2014). Costunolide and dehydrocostuslactone, two natural sesquiterpene lactones, ameliorate the inflammatory process associated to experimental pleurisy in mice. Eur J Pharmacol.

[56] Zheng H, Chen Y, Zhang J, Wang L, Jin Z, Huang H (2016). Evaluation of protective effects of costunolide and dehydrocostuslactone on ethanol-induced gastric ulcer in mice based on multi-pathway regulation. Chem Biol Interact.

[57] Akpinar E, Halici Z, Cadirci E, Bayir Y, Karakus E, Calik M (2014). What is the role of renin inhibition during rat septic conditions: Preventive effect of aliskiren on sepsis-induced lung injury. Naunyn Schmiedebergs Arch Pharmacol.

[58] Ye B, Chen X, Chen Y, Lin W, Xu D, Fang Z (2023). Inhibition of TAK1/TAB2 complex formation by costunolide attenuates obesity cardiomyopathy via the NF-κB signaling pathway. Phytomedicine.

[59] Hao J, Shen X, Lu K, Xu Y, Chen Y, Liu J (2023). Costunolide alleviated DDC induced ductular reaction and inflammatory response in murine model of cholestatic liver disease. J Tradit Complement Med.

[60] Liu W, Yang W, Niu R, Cong L, Jiang M, Bai G (2023). Costunolide covalently targets and inhibits CaMKII phosphorylation to reduce ischemia-associated brain damage. Phytomedicine.

[61] Chen Z, Zhang D, Li M, Wang B (2018). Costunolide ameliorates lipoteichoic acid-induced acute lung injury via attenuating MAPK signaling pathway. Int Immunopharmacol.

[62] Liu A, Zhang Y, Xun S, Zhou G, Hu J, Liu Y (2023). Targeting of cold-inducible RNA-binding protein alleviates sepsis via alleviating inflammation, apoptosis and oxidative stress in heart. Int Immunopharmacol.

[63] Zhang W, Chen H, Xu Z, Zhang X, Tan X, He N (2023). Liensinine pretreatment reduces inflammation, oxidative stress, apoptosis, and autophagy to alleviate sepsis acute kidney injury. Int Immunopharmacol.

[64] Eliza J, Daisy P, Ignacimuthu S (2010). Antioxidant activity of costunolide and eremanthin isolated from Costus speciosus (Koen ex. Retz) Sm. Chem Biol Interact.

[65] Üstündağ H, Doğanay S, Kalındemirtaş FD, Demir Ö, Huyut MT, Kurt N (2023). A new treatment approach: melatonin and ascorbic acid synergy shields against sepsis-induced heart and kidney damage in male rats. Life Sci.

[66] Andrews P, Azoulay E, Antonelli M, Brochard L, Brun-Buisson C, de Backer D (2006). Year in review in intensive care medicine 2005 I acute respiratory failure and acute lung injury, ventilation, hemodynamics, education, renal failure. Intensive Care Med.

[67] Romanovsky A, Morgan C, Bagshaw SM (2014). Pathophysiology and management of septic acute kidney injury. Pediatr Nephrol.

